# Roles of Cross-Membrane Transport and Signaling in the Maintenance of Cellular Homeostasis

**DOI:** 10.1007/s12195-016-0439-6

**Published:** 2016-04-28

**Authors:** Inchul Cho, Mark R. Jackson, Joe Swift

**Affiliations:** Wellcome Trust Centre for Cell-Matrix Research, University of Manchester, Oxford Road, Manchester, M13 9PT UK

**Keywords:** Plasma membrane, Endoplasmic reticulum, Nuclear envelope, Nuclear pore complex, LINC complex, Nuclear lamina, Mechanotransduction, Molecular chaperone, Stress response

## Abstract

Organelles allow specialized functions within cells to be localized, contained and independently regulated. This separation is oftentimes achieved by selectively permeable membranes, which enable control of molecular transport, signaling between compartments and containment of stress-inducing factors. Here we consider the role of a number of membrane systems within the cell: the plasma membrane, that of the endoplasmic reticulum, and then focusing on the nucleus, depository for chromatin and regulatory centre of the cell. Nuclear pores allow shuttling of ions, metabolites, proteins and mRNA to and from the nucleus. The activity of transcription factors and signaling molecules is also modulated by translocation across the nuclear envelope. Many of these processes require ‘active transportation’ against a concentration gradient and may be regulated by the nuclear pores, Ran-GTP activity and the nuclear lamina. Cells must respond to a combination of biochemical and physical inputs and we discuss too how mechanical signals are carried from outside the cell into the nucleus through integrins, the cytoskeleton and the ‘linker of nucleo- and cyto-skeletal’ (LINC) complex which spans the nuclear envelope. Regulation and response to signals and stresses, both internal and external, allow cells to maintain homeostasis within functional tissue.

## Introduction: Compartments Within Cells

**Figure 1:**
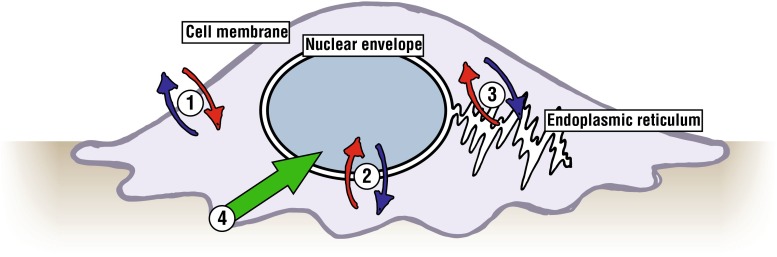
A selection of key compartments within the cell and the mechanisms that allow signaling and transportation across the membranes that separate them. (1) The plasma membrane allows transport of water and small molecules, endocytosis of nutrients and exocytosis of waste and secreted cell products. (2) Pores within the nuclear envelope allow ingress of metabolic molecules and signaling factors and egress of mRNA. (3) The endoplasmic reticulum (ER) is the cellular centre for biosynthesis: mRNA is translated by ribosomes, converting amino acid precursors into proteins that are folded by molecular chaperones. (4) Biochemical and mechanical signals must be transmitted from receptor and integrin complexes at the cell periphery through to the nucleus where they can regulate cellular responses and transcriptional programs.

The plasma membrane is one of the most important evolutionary innovations as it retains molecules within the cell at sufficient concentrations to allow the complex biochemical reactions that constitute life. Besides the plasma membrane that defines the cell, other important membrane structures include the nuclear envelope (NE), which encloses chromatin within the nucleus,^14^ and the membrane of the endoplasmic reticulum (ER), where a subset of proteins and lipids are manufactured (see Fig. 1). Compartmentalization facilitates the maintenance of intracellular homeostasis, for example by allowing ‘high-stress’ conditions, such as the presence of unfolded proteins^72^ or reduction/oxidation chemistry,^96^ to be contained within isolated environments and thus regulate and protect overall functionality of the cell. However, cells cannot be completely isolated with respect to their environments and similarly organelles must be able to communicate with each other in order to form a greater functional unit.^77^ Membranes must therefore be selectively permeable to allow transport of molecules and permissive to the propagation of the signals necessary for dynamic cell regulation. In addition, because transport may sometimes be required in opposition to a gradient of concentration, many processes require ‘active transport’ mechanisms that effectively pump required molecules across a membrane. This review will consider the roles and permittivity of the plasma membrane, the ER and the nuclear envelope (which is contiguous with the ER membrane), drawing on examples of where these structures help to maintain cellular homeostasis. It will also discuss how biochemical and mechanical signals can be transduced from the extracellular environment through to internal regulatory mechanisms within the cell and in particular the nucleus, thus enabling the cell to continually adapt to the demands of function and the environment.

## The Plasma Membrane

The plasma membrane (also referred to as the cell or cytoplasmic membrane) is a selectively permeable phospholipid bilayer that separates and protects the cell’s interior from the surrounding environment. The maintenance of intracellular homeostasis requires regulated uptake and release of ions and other molecules, along with the bidirectional transmission of signals. To achieve this, the plasma membrane is embedded with channel and receptor proteins that respond to chemical and physical cues. Cell surface receptors transduce external signals, while integrins and other adhesion proteins facilitate cell attachment and enable the interrogation of the physical compliance of the extracellular environment. These surface proteins are therefore able to relay both mechanical and biochemical inputs into a range of intracellular signaling pathways, allowing for the continual sampling of and adaption to environmental changes. Precise spatial and temporal control of such signaling, for example through negative feedback mechanisms, is critical for the maintenance of homeostasis.

### Interactions Between Cells and the Extracellular Matrix

The ECM contains a complex network of proteins and glycosaminoglycans. Extracellular collagens are the most abundant proteins in the human body, forming triple-helical structures that can assemble into fibers and networks that define the mechanical properties of our tissues.^86^ The ECM is continuously remodelled to meet homeostatic tissue requirements, as resident cells secrete new matrix and enzymes that can modify or degrade the existing matrix.^12^ Additional constituents of the ECM include fibronectin, a ~ 440 kDa, glycosylated (sugar-modified) protein that binds integrins, and proteoglycans (including highly glycosylated proteins such as decorin, biglycan and lumican), which are able to sequester growth factors. Integrins are responsible for cell–matrix binding but also sense information about attachment and local properties of the ECM. Variation within integrin complexes—different dimeric combinations of an *α* and a *β* subunit—determines cellular preference for specific ECM composition. Integrins transduce signals across the plasma membrane to the cytoskeleton* via* a complex of talin, vinculin and other proteins, forming structures called ‘focal adhesions’ (FAs). Signaling cascades are initiated by conformational changes that occur within FAs as integrins engage and pull against the ECM.^7^,^17^ Further to integrin attachment, additional matrix-associated signaling may be employed, for example following the functionalization of the ECM with growth factors. Such factors may specify tissue development, such as bone morphogenic proteins (BMPs, which drive the formation of bone and cartilage)^9^ and nerve growth factors (NGFs),^66^ or initiate particular cell responses, for example, tumour necrosis factors (TNFs, which promote cell death).^10^ The concerted action of cell surface receptors and integrins is necessary for the maintenance of the equilibrium that exists between cells and their respective extracellular environment.

### Transport Across the Plasma Membrane

The internal portion of the plasma membrane is hydrophobic and therefore acts as a barrier to ion and water transport. This allows cells to control the uptake and release of factors through the regulation of channel complexes, which perforate the plasma membrane. To facilitate the movement of water across the membrane, cells express water channels named ‘aquaporins’. To date, thirteen human aquaporins, often exhibiting tissue specific expression, have been identified.^18^ The selective water permeability of an aquaporin can be explained by its structure: an aquaporin is a homo-tetramer with each subunit traversing the plasma membrane. Six transmembrane *α* helices contribute to the water selective central pore^81^ by forming an aromatic/arginine constriction site. This site effectively excludes protons and re-orders surrounding water molecules.^19^ Additionally, the intracellular second and extracellular fifth loops of the protein contain an Asn-Pro-Ala motif sufficient to preclude the passage of ions, thereby allowing the preferential transport of water molecules.^102^

Many charged ions enter the cell by passively diffusing through gated channels, which are regulated by ligand binding or differences in potential across the membrane. The movement of molecules against a concentration gradient requires a process known as ‘active transport’. As energy is required for active transport, the establishment of an electrochemical gradient (a gradient of electric potential and concentration) during primary active transport can improve efficiency. Subsequent processes that utilize this electrochemical gradient, referred to as secondary or tertiary active transport, often employ a carrier that travels down its gradient of concentration. If the molecule being carried travels in the same direction as the carrier, the process is referred to as ‘symport’; where the two molecules travel in opposing directions, the process is referred to as ‘antiport’. For example, the active import of potassium and exclusion of sodium ions by the transmembrane Na^+^/K^+^-ATPase is important in several processes, including preservation of intracellular homeostasis.^53^ Differences in potassium, chloride and sodium ion concentrations are primarily responsible for establishing the electrochemical potential across the membrane. To resist unwanted diffusion of water into the cell, the plasma membrane must allow for application of an osmotic pressure for appropriate osmoregulation. Indeed, modest disruption of this regulation by subjecting chondrocytes to external changes in osmotic potential, a model of the pathological degradation of cartilage, has been shown to initiate cell responses including chromatin remodelling.^38^ Thus, maintenance of ionic concentration across the membrane through control of water and ion transport is crucial to cell integrity and function.

Bulk transport across the membrane (import, endocytosis; and export, exocytosis) is achieved using enclosed vesicles. Vesicles are formed as the cell imposes curvature and then ‘pinches off’ regions of membrane. The most well understood of such mechanisms involve clathrin-coated vesicles.^43^ The formation of clathrin-coated vesicles is initiated by the recruitment of the adaptin complex to a receptor following a ligand-induced conformational change. Adaptin allows the binding of clathrin proteins, which form a basket-like structure—acting as a template for vesicle formation. Finally dynamin pinches off the now spherical vesicle, allowing it to bud away from the plasma membrane. Internalization of receptors by such mechanisms can be used to tune the sensitivity of signaling pathways, potentially protecting cells from over-response to stimulation.^44^ The secretory pathway employs similar mechanisms of regulated vesicle budding and fusion. Retrograde vesicles that bud off from the Golgi apparatus use COP-I coated vesicles, whereas anterograde vesicles leaving the endoplasmic reticulum employ COP-II coating.^93^ Where necessary, this pathway is further regulated by the presence of a localization signal in the cargo protein, which directs the vesicle to the appropriate cellular compartment. The equilibrium between exocytosis and endocytosis across membranes is important for homeostasis as it ensures coat proteins and particularly lipids—a limited resource within the cell—are recycled.

## The Endoplasmic Reticulum

In addition to the plasma membrane, intracellular membranes separate functional processes into defined organelles. The endoplasmic reticulum (ER) is one such membrane-contained subcellular compartment involved in protein synthesis and post-translational modification (PTM), and encloses the initial steps of the secretory pathway.^68^ The ER also has roles in intracellular signaling by storing calcium ions, which are selectively released upon stimulation. Modulation of the concentration of this second messenger is featured in many signaling pathways including voltage sensing mechanisms, and those downstream of growth factor receptors and G-protein coupled receptors (GPCRs) present on the plasma membrane. In such pathways, ligand binding leads to activation of phospholipase C and so the production and release of inositol-1,4,5-triphosphate (IP3) from the plasma membrane. The ER membrane is decorated with IP3 receptors which act as pores following stimulation, releasing calcium stored within the ER lumen. This resulting increase in cytoplasmic calcium concentration leads to downstream events including transcription factor regulation, for example in the case of CBP (CREB binding protein).^8^

### Insights into Cross-Membrane Signaling from the Unfolded Protein Response

Proteins entering the secretory pathway are co-translationally transported into the ER lumen or inserted into its membrane. Thus, the ER houses molecular chaperones, which assist with proper protein folding, and enzymes required for PTMs, including specific glycosylations. It has been suggested that limitation of the protein load in the ER protects against age-related diseases^89^ and promotes health^57^ by enhancing the quality of protein-folding. In agreement with this notion, elevated chaperone expression was found to positively correlate with increased lifespan.^78^

Under non-equilibrium conditions where nascent proteins begin to accumulate in the ER, for example, when glycosylation and protein export is inhibited,^47^ the chaperone machinery can be overwhelmed, promoting protein aggregation. This potentially pathological accumulation of proteins leads to activation of the unfolded protein response (UPR). The UPR aims to restore homeostasis by alleviating stress associated with the build-up of unfolded proteins in a number of ways: by decreasing the overall rate of protein translation; by increasing capacity through upregulation of ER membrane and molecular chaperone proteins; and by increasing the rate of proteolytic degradation of misfolded proteins. Prolonged and unmanageable levels of stress promote activation of apoptosis. The UPR is dependent on the activity of three transmembrane receptor proteins: inositol requiring protein-1 (IRE1), protein kinase RNA-like ER kinase (PERK) and activating transcription factor 6 (ATF6). The signaling cascades initiated by these proteins reinforce each other and there is significant functional redundancy, thus ensuring a robust response.^34^ One model suggests that in the absence of misfolded proteins, the lumenal domains of each of the three receptor proteins are bound to an ER chaperone, BiP (also known as 78 kDa glucose-regulated protein, GRP-78). However, when misfolded proteins accumulate in the ER, BiP dissociates from the membrane-bound receptor proteins and binds to the exposed hydrophobic domains of misfolded proteins. Loss of inhibitory BiP binding is therefore proposed to activate the sensor proteins and so the UPR pathway. Activated IRE1 forms a homodimer which auto-phosphorylates in its cytoplasmic domain.^13^ This autophosphorylation initiates IRE1 endonuclease activity, leading to operative splicing of mRNA encoding the transcription factor XBP1.^87^ Subsequent translocation of XBP1 protein into the nucleus activates the transcription of proteins that can assist in the alleviation of stress.^104^ Like IRE1, PERK also forms a homodimer upon activation that subsequently undergoes autophosphorylation.^1^ Active PERK phosphorylates eukaryotic translation initiation factor 2*α* (eIF2*α*), inhibiting its interaction with eIF2*β*. This process globally inhibits translation, thus decreasing the overall load on the ER, while allowing for the selective up-regulation of stress-response proteins, such as transcription factor ATF4.^97^ Subsequent translocation of ATF4 into the nucleus causes up-regulation of protein-folding enzymes and the transcription factor CHOP, which in turn activates the pro-apoptotic protein GADD34. Programmed cell death, however, is a last resort: the pathway has built-in negative feedback, with GADD34 binding phosphatase-1C to accelerate dephosphorylation of eIF2*α*. Loss of BiP binding from ATF6 exposes an export signal, causing translocation of the protein to the Golgi where an inhibitory domain is cleaved, releasing the active fragment;^83^ this fragment is transported to the nucleus where it upregulates proteins from the ER-associated protein degradation (ERAD) pathway.

The UPR illustrates the important role of intracellular membrane structures in isolating processes, in this case the build-up of potentially toxic unfolded proteins, but also the need to rapidly transduce signals across membranes and throughout the cell. Proteins such as IRE1, PERK and ATF6 can act as signaling conduits as they span the ER membrane. These pathways are also subsequently dependent on release and translocation of an active factor into the nucleus, which requires a mechanism to traverse a second membrane, the nuclear envelope. Thus, the UPR—essential for maintenance of homeostasis—represents a complex response relying upon the coordination of multiple signaling modalities acting across different subcellular compartments.

## The Nuclear Envelope

**Figure 2:**
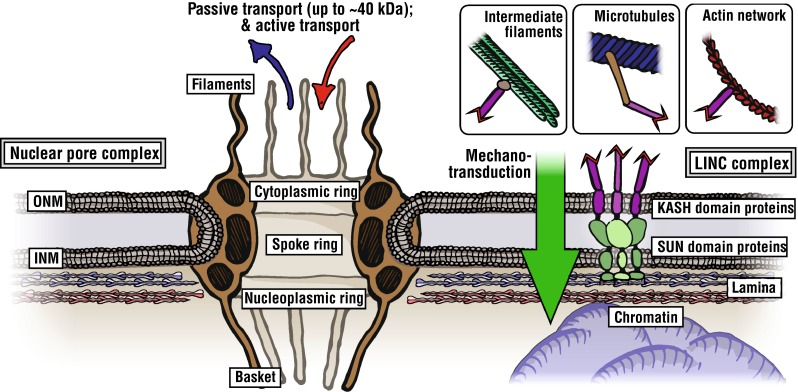
The nuclear pore and ‘linker of nucleo- and cyto-skeleton’ (LINC) complexes are shown embedded within the phospholipid bilayer of the nuclear envelope (composed of outer and inner nuclear membranes, ONM and INM). The nuclear pore complex has three ring moieties: nucleoplasmic ring; spoke ring and cytoplasmic ring. The spoke ring forms a central channel which FG Nups (phenylalanine-glycine repeat nucleoporins) are thought to align with. Within the perinuclear space, there are SUN domain containing proteins which couple networks of cytoskeletal proteins, such as actin filaments and microtubules, and nucleoplasmic structural proteins, principally lamins A and B in the nuclear lamina.

The nuclear envelope (NE) effectively segregates the genetic information encoded within DNA from the cytoplasm. Containing DNA within an enclosed double phospholipid bilayer allows for additional control of signal transduction and gene expression by regulation of the passage of transcripts and proteins, as well as protecting the genetic information. The inside of the NE is lined by the nuclear lamina, a filamentous protein structure that lends mechanical robustness to the nucleus. The nuclear envelope is perforated by large multimeric protein structures called nuclear pore complexes (NPCs) (see Fig. 2). These pores interact with the lamina and proteins embedded within the NE, and mediate the transport of proteins and mRNA. The NE often appears wrinkled with invaginations into the nuclear body. It has been proposed that this wrinkling may allow the nucleus to change its shape (with associated changes in volume to surface-area ratio) in response to external perturbations.^92^

### The Nuclear Pore Complex

The nuclear pore complex (NPC) allows transport of macromolecules between the nucleus and cytoplasm. The assembled NPC is large, approximately 125 MDa in humans,^69^ with in-the-order of thousands of such complexes found per nucleus.^21^ Structurally, NPCs comprise of three layers of nucleoporin proteins (‘Nups’), with each layer consisting of a ring with eight-fold rotational symmetry defining a central channel (Fig. 2). There are approximately thirty different Nups, which occur in multiple copies within the NPC. Nups themselves are highly heterogeneous and some are able to form smaller complexes. The Nup107 complex consists of seven proteins that form a Y-shaped structure, which assemble to form the octameric ring of the NPC.^39^ The Nup107 complex appears to be essential for the integrity of the NPC, providing a scaffold for other Nups.^98^ The inner surface of the nuclear pore is lined with ‘FG Nups’—nucleoporins containing intrinsically disordered domains of phenylalanine-glycine repeats—which are thought to act as a selective barrier to macromolecules (>40 kDa).^75^

### Transport Through Nuclear Pores

Ions, such as calcium, are able to diffuse through the nuclear pores but large molecules, proteins and RNAs cannot cross the nuclear envelope without active transportation. The nuclear pores allow molecules of up to approximately 40 nm in diameter to be transported across the NE.^63^ Molecules up to 5 kDa are able to passively diffuse through NPCs, but as protein size increases diffusion becomes slower. Above 40 kDa, proteins can no longer passively diffuse and must instead be actively transported. Nuclear resident proteins, such as histones and polymerases, are synthesised in the cytoplasm and must therefore be shuttled into the nucleus. Conversely, some nucleic acids, such as tRNA and mRNA, are transcribed in the nucleus and must be exported for function. Furthermore, the regulated shuttling of proteins between nucleus and cytoplasm represents an important signaling modality. Hence there is a need for selective, regulated, bidirectional transport across the NE.

A ‘nuclear localization signal’ (NLS) is a short sequence of lysine and arginine residues that marks a protein for transportation to the nucleus.^40^ Proteins containing the ‘classical’ NLS sequence (KKKRK) are bound by importin *α* and subsequently importin *β*, which are adaptor and receptor proteins respectively;^90^ proteins with ‘non-classical’ NLS sequences may bind to importin *β* directly. The importins belong to a larger family of nuclear transport proteins called karyopherins. The selectivity of translocation is achieved through interactions between cargo-karyopherin complexes and the FG Nups that line the walls of the nuclear pores.^25^ The size, complexity and dynamic nature of the NPC has made it difficult to study with conventional imaging methods^37^ and consequently the native structure of the FG Nups is poorly understood, as is the stepwise mechanism of nuclear import and export. Nonetheless, a recent bioinformatics study of evolutionarily conserved structures has identified that FG Nups maintain specific distributions of FG motifs, polar and charged residues.^65^ Several models have been proposed to explain transport through the nuclear pores: in the ‘selective phase’ model of translocation, the FG Nups are proposed to interact with one another to form a gel-like structure within the pore complex. Proteins that can interact with the FG repeats, such as transport receptors, are then able to locally melt the hydrogel and so pass through the pore.^36^,^70^ Other theories include a ‘reduction of dimensionality’ model, in which selected cargoes are directed through an FG-rich channel as opposed to a diffusive pathway;^64^ ‘virtual gate’^74^ and ‘reversible collapse’^50^ models posit a ‘brush-like’ action of FG domains in excluding inert molecules, with the latter model also proposing that contraction of the FG domains acts to further drive selected cargoes through the pore. Another study has suggested that FG Nups can be classified into two regions that support distinct transport pathways.^103^

The process of nuclear export is conceptually similar to import: but requires that cargoes contain a ‘nuclear export signal’ (NES), which is comprised of a short sequence of hydrophobic amino acids. The NES is recognized by karyopherins that mediate nuclear export, known as exportin proteins. The maintenance of translocation processes requires karyopherins to be continuously cycled in and out of the nucleus. To facilitate this, importin *α*, for example, is recognized by the exportin CAS (‘cellular apoptosis susceptibility’ protein).^61^ As transport across the nuclear membrane is an active process, it requires an input of energy—this is provided by the Ran-GTP cycle.

### The Ran-GTP Cycle

In transport processes, the destination for delivery must be distinguishable from the point of origin. This is achieved in nuclear import and export by the spatial separation of Ras-related nuclear protein (Ran protein) bound to either guanosine diphosphate (GDP) or guanosine triphosphate (GTP).^71^ This differential is established by two key proteins: Ran-GTPase activating protein (Ran-GAP), which is retained on the cytoplasmic side of the NPC; and regulator of chromosome condensation 1 (RCC1), which is bound to chromatin and so remains nuclear.^59^ Ran is a small protein (25 kDa) and so is able to diffuse through the nuclear pores, but in the cytoplasm Ran-GAP stimulates the GTPase activity of Ran, causing hydrolysis of bound GTP. Conversely, RCC1 in the nucleus is a ‘guanine nucleotide exchange factor’ (GEF), which exchanges GDP bound to Ran for GTP. In this way, the two proteins work in concert to establish a relatively low cytoplasmic and high nuclear concentration of Ran-GTP.

Karyopherins, such as the importins, bind to cargo proteins and enter the nucleus through the nuclear pores. Inside the nucleus, importin *β* binds to Ran-GTP, causing a conformation change and release of the cargo. The importin-Ran-GTP complex then exits the nucleus, and once in the cytoplasm the GTP is hydrolysed freeing the importins to continue the transport cycle. Nuclear export is driven by the same Ran-GTP gradient: exportins such as CAS bind both Ran-GTP and a cargo protein (such as importin *α*, as described earlier) and this complex exits the nucleus through the pore. Subsequent hydrolysis of the GTP triggers the release of Ran-GDP and the cargo protein. Ran-GDP is returned to the nucleus through interaction with NTF2 (nuclear transport factor 2), where it is recharged with GTP—thus completing a cycle of translocation.

### Regulation of Nuclear Transport Under Stress Conditions

Just as the functionality of the ER is protected by the UPR, the homeostasis of protein folding in the cytoplasm is maintained by a pathway called the ‘heat shock response’. Although nominally associated with thermally-induced denaturation, the response also mitigates protein damage from oxidative, chemical, mechanical and disease-associated stresses. The build-up of unfolded proteins causes complexes of molecular chaperones (comprised of heat shock proteins, HSPs) to dissociate, releasing the transcription factor heat shock factor 1 (HSF1). The HSPs manage their unfolded ‘client’ proteins by isolating them, refolding them or marking them for proteolysis, while HSF1 translocates to the nucleus to activate the transcription of additional HSPs.^32^ During heat shock, importin *β*-mediated nuclear import is suppressed, but shuttling of heat shock protein 70 (HSP70) to the nucleus is increased through interaction with ‘Hikeshi’ protein.^45^ The activity of HSP70 is based on a conformational change powered by an ATP/ADP cycle (adenosine tri-/di-phosphate, ‘energy carriers’ related to GTP/GDP) that is thought to promote protein refolding; Hikeshi selectively transports the ATP-bound form into the nucleus. The number of HSP70 proteins in the cell is typically high, but its functionality in the nucleus is poorly understood: HSP70 has been implicated in the maintenance of cell viability, and to negatively feedback on HSF1,^45^ whilst also directing misfolded proteins to the nucleolus^58^ and assisting in single-strand DNA damage repair.^46^

## The Nuclear Lamina

The proteinaceous lamina structure that lines the inside of the nuclear envelope is composed primarily of intermediate filament lamin proteins. In mammals, the family is divided into two types. Type-A lamins are encoded by a single gene, *LMNA*, which is alternatively spliced to produce two main isoforms, lamins A and C; type-B lamins are coded for by *LMNB1* and *LMNB2* producing lamin B1 and B2 proteins, respectively. The lamins share conserved structural features typical of intermediate filaments: a globular tail domain; a rod-like coiled-coil domain that facilitates assembly into parallel dimers; and an immunoglobulin-like head domain (which contains an NLS to direct lamins to the nucleus). Lamins A, B1 and B2 are post-translationally modified to append a hydrophobic farnesyl moiety to the C-terminus. This modification potentially serves to localize lamins B1 and B2 to the inner nuclear membrane, but is proteolytically cleaved from the mature form of lamin A.^26^

Lamin dimers can interact head-to-tail, assembling further in a staggered, anti-parallel manner to form filaments and higher-order meshwork structures.^26^ Although several domains within lamins have been studied crystallographically, and high resolution electron and light microscopies have provided insight into higher-order lamina structures, there remains an intermediate length scale that is poorly understood. The study of lamina organization has been complicated by issues in comparing *in vitro* model systems with the architecture found in cells, where the presence of chromatin and other lamin-binding proteins likely influences assembly. Studies have shown, for example, that A- and B-type lamins can copolymerize when mixed at high concentrations,^41^ but high-resolution microscopy studies in cells have instead observed interacting but distinct networks of the two lamin families.^84^

Study of the nuclear lamina has been motivated by the discovery of a range of disease conditions linked to mutations or misregulation of A-type lamins, referred to as ‘laminopathies’.^101^ Despite the widespread somatic expression of lamin A, the manifestations of these disorders can be distinct and often tissue-specific: in heart (dilated cardiomyopathy, DCM);^3^ in muscle (Emery–Dreifuss muscular dystrophy, EDMD);^2^ in adipose tissue (familial partial lipodystrophy, FPLD);^82^ or, rarely, in neurological disorders. In Hutchinson–Gilford progeria syndrome (HGPS), a truncated form of lamin A that cannot be correctly post-translationally processed causes a ‘premature ageing’ phenotype,^20^ often resulting in heart failure ensuing from atherosclerosis. The aetiology of these diseases is not fully understood, but are thought to occur as a consequence of disruption of the homeostatic regulation of nuclear mechanics and gene expression,^16^ discussed in the following sections. A number of mouse models have been developed to investigate the role of lamins, with complete knockout of A-type lamins causing defects in heart and adipose tissue. Relatively few disorders have been associated with misregulation of B-type lamins (rare cases exhibit lipodystrophy and leukodystrophy), perhaps as other phenotypes have not yet been attributed to lamin B mutation, or because loss of function proves to be lethal during embryogenesis—as observed following knockout of murine B-type lamins.^26^ In this model, embryos exhibited defects in multiple organs, in particular during brain development, where neuronal migration was compromised.^42^

### Lamins Define Nuclear Mechanical Properties that are Matched to the Physical Demands on Tissues

Despite similarities in their structure and assembly, A- and B-type lamins impose distinct characteristics on the mechanical properties of nuclei.^31^,^48^,^85^,^92^ Nuclei rich in A-type lamins are stiffer and exhibit viscous deformation; whereas high levels of B-type lamins confer elasticity. Stiff tissues often have load-bearing or protective roles and are therefore subjected to repeated, sometimes rapid, deformation. In these situations, a lamina rich in A-type lamins provides protection to chromatin by acting as a ‘molecular shock absorber’.^15^,^91^ It has been known for many years that the expression patterns of lamins are highly tissue specific.^4^ Stiff tissues, such as bone, muscle and cartilage, have cells with nuclei rich in A-type lamins; non-load bearing soft tissues, such as brain and marrow have a greater relative proportion of B-type lamins.^92^ The mechanical properties of nuclei therefore appear well matched to the functions and demands of the mature tissues in which they reside.^91^ Although not a universal explanation, these observations perhaps hint at why many of the phenotypes associated with loss of lamin A function are primarily manifested in stiff tissues (in both the clinical and experimental context). However, in tissues less-dependent on mechanical robustness, a stiff nucleus could be disadvantageous. White blood cells/leukocytes, which are required to deform elastically during circulation and infiltration into tissue, have elevated levels of B-type lamins and hence a more elastic nucleus.^85^ Furthermore, the composition of the lamina has also been implicated in determining the invasiveness of cancer cells,^31^,^100^ suggesting nuclear stiffness as an important factor in migratory capacity.^31^,^76^ These studies highlight the role of the nuclear lamina in cell and tissue-scale mechanical homeostasis.

### Lamin Interactions with LINC Complexes and Chromatin

Lamins are involved in a broad range of interactions within the nucleus: they can bind to nuclear envelope proteins, components of signaling pathways and transcription factors;^33^ they can direct the distribution of NPCs;^29^ and are responsible for anchoring chromatin—in particular, transcriptionally inactive heterochromatin—to the nuclear periphery.^26^ The linker of nucleo- and cyto-skeleton (LINC) complexes connect the nuclear lamina to the cytoskeleton (Fig. 2).^73^ Lamin proteins interact with SUN-domain containing proteins in the inner nuclear membrane, which in turn bind the KASH domains of nesprins, spanning the perinuclear lumen and extending from the outer nuclear membrane to interact with actin, microtubules (through intermediary kinesins) and intermediate filaments (through plectin).^88^ Thus, there is an apparently continuous, interconnected succession of structural proteins linking FAs at the cell membrane, through cytoskeletal components and the LINC complexes to the lamina and chromatin in the nucleus.^30^ Such mechanical linkages to the lamina are thought to have a role in signaling, discussed in the following section, but there is also evidence of load-bearing functionality. During cell migration, for example, the nucleus is pulled by ‘TAN lines’ (transmembrane actin-associated nuclear lines), composed of an interconnected succession of actin, LINC complex and nuclear lamina.^51^ Mutant lamin A proteins, defective in anchoring TAN lines, have been shown to cause striated muscle diseases,^24^ evidencing their structural role and suggesting a central importance to the maintenance of mechanically-loaded tissue.

Interactions between chromatin, the lamins and other proteins associated with the lamina impose spatial organization within the nucleus. The human genome contains more than a thousand ‘lamina-associated domains’ (LADs), which are genomic regions characterized by inactive/repressed or low-transcription genes and heterochromatic histone modifications that are located at the nuclear periphery.^27^ Knockdown of lamins and lamin-associated proteins has been shown to disrupt chromatin organization.^26^ Accordingly, changes in lamin-chromatin interactions, genome organization and DNA methylation profiles have been observed in laminopathic cells.^55^ The intranuclear location of chromatin domains was found to change upon gene activation during development, with dependency on the composition of the nuclear lamina.^95^ This is suggestive of a role for lamin-dependent gene regulation during differentiation, although the necessity of specific proteins is possibly limited by some degree of functional redundancy, as lamin knockout mice are still able to form all somatic tissues. Thus, the lamina likely additionally promotes homeostasis through its influence upon gene regulation and genome organization.

Other important processes regulated by lamina interactions—where failure could also contribute to the laminopathic phenotype—include the regulation of adult stem cell populations^80^ and NF-*κ*B mediated inflammatory responses.^60^ The lamina has also been implicated in DNA damage repair pathways, with cells expressing defective progeria-associated mutant forms of lamin-A favoring error-prone non-homologous end joining (NHEJ) repair of double-strand DNA breaks.^105^

## Mechanotransduction to the Nucleus

Adherent cells are able to ‘feel’ and respond to the geometric constraints and mechanical properties of their surroundings. Mesenchymal stem cells (MSCs) can be driven towards different fates by directing their shapes, with highly spread vs. restricted cells tending towards osteogenic and adipogenic differentiation, respectively.^54^ MSCs also exhibit distinct phenotypes and lineage commitment when cultured on hydrogels of different stiffnesses.^23^ Modulation of cell shape and substrate stiffness alter the distribution of forces in the cytoskeleton and change protein conformations in the FA complexes. These conformational changes can affect affinity for particular binding partners, and hence initiate specific signaling cascades within the cell. For example, extension under tension of the FA-associated protein pCas130 leads to increased phosphorylation and subsequent activation of Rap1 signaling.^79^ ‘Mechanotransduction’ processes—ways in which mechanical changes are converted into biochemical signals—remain a subject of broad experimental inquiry. A range of pathways have been identified, including: signaling cascades from conformational sensitivity within FAs; mechano-responsive ion channels that allow rapid transport across the plasma membrane;^52^ and mechano-sensitive cytoplasmic-to-nuclear translocation of transcription factors, such as serum response factor (SRF)^11^ and yes-associated protein 1 (YAP1).^22^ It is likely that these pathways can act in concert, both to provide a broad dynamic range of activity and to give robustness through functional redundancy. Nonetheless, some questions remain, such as how mechanical inputs with little spatial coherence can be directed to regulate very specific actions, such as in modulating the transcription of particular genes in the nucleus to direct cell differentiation. Another potential avenue for investigation is the connection between mechanical input and stress response pathways. For example, proteins related to HSP70 are expressed in response to mechanical input (such as in muscles following exercise), but the functional mode of this presumably homeostasis-maintaining ‘protective’ action is not well understood.^49^

It is believed that FAs, the cytoskeleton, LINC complexes and lamina can act as a pathway of ‘mechano-transmission’, effectively a series of mechanical linkages carrying inputs from outside the cell, across membranes and to the nucleus. The composition of the nuclear lamina has been demonstrated to actively respond to the mechanical environment outside the cell, with MSCs cultured on stiff substrates increasing the amount of lamin A in their nuclei, making them correspondingly stiffer. This process is regulated by a mechano-sensitive phosphorylation mechanism: a stiffer substrate drives a more tensile cell phenotype; this tension is propagated to the nucleus where phosphorylation of lamin A is correspondingly suppressed; decreased phosphorylation promotes lamin assembly into the lamina.^6^,^92^ Nuclear stiffening in response to mechanical input has also been demonstrated in isolated nuclei, using magnetic tweezers to apply forces to LINC complexes through nesprins. In these experiments, dependence was shown on lamin and phospho-regulation of emerin, a lamin-binding protein associated with the inner nuclear envelope.^28^

The connection between FAs and the nucleus means that extracellular and nuclear mechanics can couple together to direct cell fate. So, for example, osteogenic differentiation is amplified when MSCs are cultured on stiff hydrogel substrates and the nucleus is additionally stiffened through overexpression of lamin A.^92^ Other work has demonstrated an outside-in linkage between cell geometry, regulation of cyto- and nucleo-skeletal organization, nuclear mechanics, and control of chromatin modifications in embryonic stem cells.^94^

## Conclusions

In order to maintain homeostasis in living tissue, cells must be robust enough to withstand or adapt to demanding conditions: these include the stresses brought about by the function of their own internal chemistry, and the external stresses imposed by the environment. Membranes make an important contribution to maintaining this robustness, providing containment and separation for processes such as protein modification and folding in the ER, and chromatin regulation in the nucleus. Signaling across membranes is also crucial to ensuring cell adaptability. This was exemplified here in the case of the regulation of molecular chaperones in response to internal stresses, and mechanotransduction pathways leading to nuclear remodelling and protection of the nucleus in the case of environmentally-applied, mechanical stresses.

It is perhaps appealing to attempt to classify the roles of certain cellular components, such as the membranes, as being either singularly chemical or mechanical in nature. A longstanding knowledge of cellular functionality has been based on biochemical approaches, considering biological processes as pathways of intermolecular interactions with spatial and conformational regulation. These methods have recently been supplemented by a recognition of physical and mechanical factors. However, physical and biochemical pathways are likely to have overlapping or complementary functions, and perhaps rely on the same components: a molecular chaperone, for example, may not distinguish between a client molecule unfolded by chemical, thermal or mechanical stress.^5^

We have discussed where mechanical modes are essential to the maintenance of cell homeostasis in active tissue, and where they can complement or amplify biochemical signaling pathways. Indeed, physical signaling pathways may offer unique advantages: mechanical processes can be extremely rapid, with signal transmission from plasma membrane to nucleus occurring within milliseconds.^99^ Similarly, it has been shown that signal transducing protein Src kinase (sarcoma kinase) is activated 40 times faster in response to mechanical stimulus than with biochemical activation through the epidermal growth factor receptor,^56^ perhaps indicative of a necessity for rapid response to mechanical stress.

Despite growing interest in the contribution of mechanical and chemical factors to the maintenance of homeostasis in tissue, important questions remain. These include: (1) Are there additional, undiscovered mechanisms of mechano-sensing, signal transmission and transduction? Novel technologies such as intramolecular tension reporters^35^ and measurements of protein conformation^62^,^92^ may aid in the identification of force sensors and downstream signaling components. (2) How can mechanical and biochemical information be integrated to allow for a specific and coordinated response to diverse stimuli? Possible mechanisms may include chromatin remodelling to modulate the action of biochemical signaling,^67^ or transduction of inputs into more general ‘signaling hubs’, such as the major intracellular kinases. (3) What are the differing effector processes following chemical/mechanical stimulation? Responses described to date include nuclear, cytoskeletal and FA remodeling, and ECM modification but may further include activation of other pathways involved in homeostasis, for example in modulating cellular stress response pathways. (4) Finally, as the biochemical and mechanical pathways that enable tissues to maintain homeostasis are better understood, can they be used to target the pathologies associated with deteriorating homeostatic balance, both in disease and ageing, and could they inform advances in regenerative medicine?
